# Food and nutritional security requires adequate protein as well as energy, delivered from whole-year crop production

**DOI:** 10.7717/peerj.2100

**Published:** 2016-07-12

**Authors:** Graeme D. Coles, Stephen D. Wratten, John R. Porter

**Affiliations:** 1 Bio-Protection Research Centre, Lincoln University, Lincoln, New Zealand; 2 Department of Plant and Environmental Sciences, University of Copenhagen, Copenhagen, Denmark; 3 Natural Resources Institute, University of Greenwich, London, United Kingdom

**Keywords:** Agroecology, Forage utilisation, Food costs, Nutrition, Whole-year production, New Zealand, Food access, Food security

## Abstract

Human food security requires the production of sufficient quantities of both high-quality protein and dietary energy. In a series of case-studies from New Zealand, we show that while production of food ingredients from crops on arable land can meet human dietary energy requirements effectively, requirements for high-quality protein are met more efficiently by animal production from such land. We present a model that can be used to assess dietary energy and quality-corrected protein production from various crop and crop/animal production systems, and demonstrate its utility. We extend our analysis with an accompanying economic analysis of commercially-available, pre-prepared or simply-cooked foods that can be produced from our case-study crop and animal products. We calculate the per-person, per-day cost of both quality-corrected protein and dietary energy as provided in the processed foods. We conclude that mixed dairy/cropping systems provide the greatest quantity of high-quality protein per unit price to the consumer, have the highest food energy production and can support the dietary requirements of the highest number of people, when assessed as all-year-round production systems. Global food and nutritional security will largely be an outcome of national or regional agroeconomies addressing their own food needs. We hope that our model will be used for similar analyses of food production systems in other countries, agroecological zones and economies.

## Introduction

Since World War II, food insecurity has been an issue concerning the world’s poorest, with the received wisdom being that such insecurity could be alleviated by eliminating local poverty (McLaren, 1974) and improving food distribution since, globally, food has historically been produced in excess of world population needs. However, future food and nutritional security has become a major concern for both rich and poor, given the present concurrence of rising human population, climate change and changing consumption habits (Porter et al., 2014). This new reality has been recognised (Graham et al., 2007; Remans et al., 2014; DeFries et al., 2015), with attention now being paid to provision of the full range of nutrients in addition to calories, and to the development of metrics describing food system resilience on an economy-by-economy basis. Cassidy et al. (2013) recognised that one important key to monitoring food security is to develop a metric for the number of people that can be nourished per unit area and per year by a particular crop or cropping system.

However, there are a number of problems in published analyses. Firstly, while the people-nourished-per-hectare metric has been applied in terms of usable calories, no similar metric has been developed for nutritious protein. Secondly, we know of no model that accounts for whole-year land utilisation, including biomass production during the period after harvest of the primary crop assessed, and before the next season’s sowing. Thirdly, in such analyses, when biomass is used for production of animal foods (such as poultry meat, eggs, pork, beef or dairy products) feed conversion factors that are now achieved in best commercial practice are not used. Fourthly, the ability of blends of crop products to provide high-quality protein efficiently (Cassidy et al., 2013; Young & Pellett, 1994) is questionable. Finally, the cost to consumers of meeting adequate daily nutrient needs (particularly protein) in relation to agroecological productivity needs to be determined.

In this paper, we show that when the above issues are addressed:

• When considered from a people-fed-per-hectare perspective, food products from dairy production are commensurate with food products from plants, in terms of meeting needs for both dietary energy and for protein;
• Such foods can supply both energy and high-quality protein to the consumer cheaply compared to plant-based foods, when ready-to-eat products are properly compared;
• Use of forage biomass produced after harvesting food crops can contribute significant extra dietary energy and high-quality protein from animal foods;
• Blends of cereal and legume flours, optimised for essential amino acid content, contain significant excesses of most dispensable amino acids, implying inefficient use of plant photosynthetic productivity. Per mole of carbon, those excess amino acids deliver similar amounts of dietary energy to carbohydrate, but in terms of plant metabolic energy, are considerably more costly to synthesise.

## Methods

### Food needs

Minimum daily energy intake required for food security lies in the range 1,800–2,000 kcal/person/day (∼7.5–8.4 MJ/person/day) (FAO Statistics Division, 2008). This amount is sufficient to meet the needs of a wealthy, sedentary, healthy adult; it is inadequate to meet those of children, growing adolescents, manual labourers or pregnant women; i.e., the majority of the human population, especially in poor countries. Adequate nutrition also requires, on average, 56 g/day of high-quality protein. A secure level of protein intake for adults is about 0.83 g/kg body mass/day (e.g., 66 g/day for an 80 kg male) and needs to include adequate provision of all essential amino acids (United Nations University, 2007). The diet must also provide adequate vitamins, minerals, essential fatty acids and dietary fibre. The elements (energy, protein, minerals, micronutrients and fibre) of an adequate diet are available from both plant and animal sources, with the exception of plant dietary fibre.

We focus on dietary energy (which may be derived from carbohydrate, lipid, protein or fermented fibre) and protein; the required intake of the latter takes account of its nutritional quality, in terms of its human digestibility and suitable amino acid profile. We assume a daily energy intake of 10 MJ (2,400 kcal), and a daily protein intake of 56 g, the biological nutritional value (BV) of which is equivalent to hen egg protein, the best suited to human needs in terms of amino acid composition. Thus, the quality of other sources of protein in terms of amino acid composition is compared against that of hen egg protein. Energy and protein contents for foodstuffs were derived from relevant information at http://nutritiondata.self.com/ which summarises USDA data. Protein BVs are taken from Akeson & Stahmann (1964).

### Production systems comparison

We analysed the total annual production of human dietary energy (MJ) and high-quality protein, corrected for its nutritional value, from crops grown on arable land for a range of crop products (Supplemental Information S1: https://figshare.com/s/ba9827a09ddb3396ce03). We assume a southern hemisphere temperate semi-maritime climate, with sufficient rainfall and irrigation water for high levels of crop growth. Under these conditions, some biomass production occurs in every month of the year. We chose this cropping framework **as an example** because reliable productivity (dry matter yield per unit area) estimates are available, but our analysis can be applied to any set of agroecological conditions in which crop yields are known. Our baseline for comparisons of the calorific content and nutritional value of different food production systems is an arable cropping system that produces only high-protein milling wheat for bread production. The assumptions we make of the baseline production system are:

• Use of a wheat cultivar from which a high yield of flour suitable for manufacture of bread is possible;
• Late-autumn sowing to achieve maximum grain yield;
• Flour extraction rate of 80%, producing 6.4 t/ha of bakers’ flour and 1.6 t/ha of offal for animal feed;
• Sowing in May (southern hemisphere late autumn), allowing after-harvest autumn production of 3.5 t/ha of brassica dry matter, used to produce milk solids from cows. The animal production achieved is credited to the milling wheat production system.

It should be noted that while data used to erect the model are derived from the range of environments found in New Zealand, these are by no means unique to that country. Similar agroecological systems may be found in Southern Australia, Southern Africa, South America, and coastal regions of the USA, the middle-to-upper latitudes of Western Europe, areas around the Black Sea, and coastal regions of East Asia.

We compared how many adults’ annual energy and quality protein needs can be met by each of the production systems (listed below, all weights as dry matter), given a calorific requirement of 10 MJ/day and 56 g/day of high-quality protein. As stated, our baseline for the comparison of systems is the energy and protein provision of bread wheat. The other whole-year crop production systems are:

• Spring-sown, winter-harvested milling maize, producing 10.5 t/ha of grain;
• Spring-sown oats, producing 6 tonnes of grain, 3 t/ha of straw suitable for forage and 5 t/ha of brassica (K Armstrong (formerly NZ Institute for Plant and Food Research Ltd) pers. comm., 2016);
• Spring-sown, summer-harvested vining peas, producing 9.5 tonnes/ha fresh weight of peas, 1 t/ha pea shaw drymatter and 8 t/ha of drymatter from summer-sown brassicas (B Snowden, Heinz-Watties Ltd, Christchurch, pers. comm., 2016)
• spring-sown field peas (*Pisum sativum*) producing 5.5 t/ha of pulses and forage oats producing 7 t/ha of drymatter. Note that pea straw is not considered a suitable forage for ruminants, even at low inclusion rates.
• Autumn-sown feed wheat, followed by summer-sown brassicas, producing 10 t/ha of grain and 3.5 t/ha of brassica
• Spring-sown, winter-harvested feed maize producing 12 t/ha of grain;
• Autumn-sown silage wheat, followed by summer-sown brassicas, producing a total of 18.5 t/ha of feed;
• Spring-sown silage maize, followed by autumn-sown Italian ryegrass, producing 29 t/ha of feed dry matter.

Crop yield information is courtesy of Dr John de Ruiter, New Zealand Institute for Plant and Food Research Ltd, unless otherwise stated. In food crop-producing systems, any autumn or winter-produced forages, and straws, stovers and grain processing wastes are allocated to milk production.

**Figure 1:**
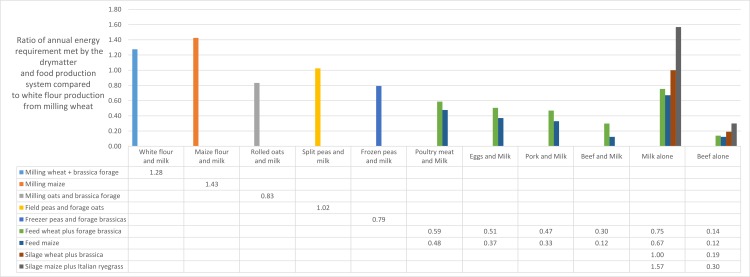
Bars represent the ratio between the numbers of people whose annual energy needs are met by the system described, and by production of milling wheat for bread (26). Gaps in the table are because not all food ingredients can be produced from any given arable production system.

**Figure 2:**
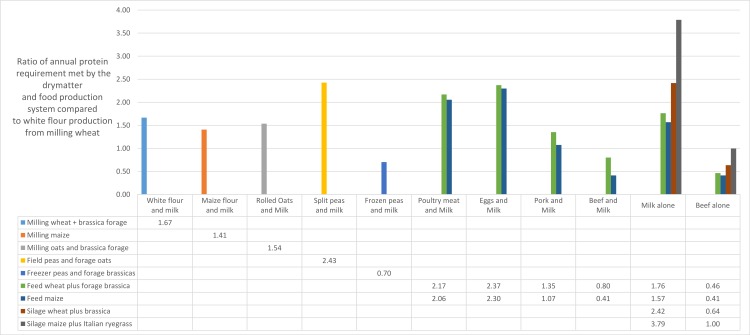
Bars represent the ratio between the numbers of people whose annual protein needs are met by the system described, and by production of milling wheat for bread (16). Gaps in the table are because not all food ingredients can be produced from any given arable production system.

The chosen cropping systems provide raw materials for the production of a range of foods or food ingredients (Figs. 1 and 2). Protein BVs used are 0.50 (white wheat flour), 0.47 (split peas, discounted by 15% for trypsin inhibitor effect), 0.75 (poultry, pork and beef) and 0.90 (milk solids) (Akeson and Stahmann, *op.cit*.). We present the data as the relative annual production per unit area (ha) of energy and protein for humans, when compared to the baseline (milling wheat alone) system (Figs. 1 and 2). In essence, we are comparing the contributions of wholly plant-based cropping systems with mixed plant–animal systems to food and nutritional security in terms of the number of persons supported for their calorific and nutritional requirements per unit area.

Some authors (e.g., Cassidy et al., 2013) claim that deficiencies in plant protein quality can be remedied by mixing food ingredients from different plant types, particularly combining cereals and legumes. To examine this assertion, we evaluated a system comprising mixtures of milling wheat and field peas produced as above. In common with other legumes, the BV of the pea protein, estimated to lie between 50% and 55% based on amino acid composition, is confounded by the presence of varying levels of trypsin (protease) inhibitors (Mariotti et al., 2001); thus the reported crude BV may be too high.

### Conversion factors

In establishing the number of people whose protein and dietary energy needs can be met from whole-year biomass production, it is important to use commercially-relevant factors for conversion of raw materials to final product; these are shown in Table 1. Attention is drawn to the dietary energy: milk solids conversion factor used, which applies to **additional** milk solids produced when cows are fed on pasture *in situ*. This factor was chosen as a compromise, because while maintenance energy requirements reduce whole-diet conversion ratio by approximately 11%, well-made supplements such as are discussed in this paper provide significant improvements in relative feed efficiency.

**Table 1:** Conversion factors used to translate crop biomass production to useful food ingredients.

*Raw material conversion*	*Live weight production (FCR: kg feed: kg liveweight)*	*Useable food ingredient (yield per kilogram live weight)*	*Notes*
Wheat to poultry meat	1.5	0.6	In commercial practice in New Zealand, whole-of-life feed conversion ratios routinely fall below 1.5 kg/kg (J Foulds, pers. comm., 2015). It should be noted that commercial feed formulations usually contain only 85% wheat or maize, with the balance made up of meat meals or, less often, plant protein sources such as soya bean meal. Small quantities of synthetic amino acids are often used.
Maize to poultry meat	1.5	0.6	
Wheat to pork	2.1	0.6	See above. In this case, FCR values are unpublished data of the senior author.
Maize to pork	2.1	0.6	
Wheat to beef	7.0	0.6	Note that the FCR used applies to the effect of using an arable crop product as a substantial supplement, not whole-of-life total diet. On the other hand, the recovery figure does not take into account the use of meatmeals for further animal production.
Maize to beef	7.0	0.6	
***Wheat or maize to milk solids***		***Yield/kg feed***	
Grain @ 12.5 MJ/kg		181 g	Budget figure for nett conversion of forage dietary energy to milk solids is 69 MJ/kg solids. New Zealand farmers are paid on the basis of the amount of protein and fat they deliver. Our calculations include a further 50% to allow for milk lactose production.
Milling offal @ 10.0 MJ/kg		144 g	
Forage @ 10.0 MJ/kg		144 g	

### Daily cost of nutrient provision

While the productivity achieved is appropriately expressed in terms of persons nourished per hectare, where available land is the limiting factor for food production, it is useful to determine the relative financial cost of meeting nutritional needs from different production systems. Conversion of raw materials to consumer-ready foods involves a variable number of unit operations of varying cost. However, these costs are summarised in the final price of the ready-to-eat product. It should be noted that the price of many such products includes an amount for the brand value associated with the producer. Therefore, the prices used in this study are derived, where possible, from products used to calculate the monthly consumer food price index generated by the New Zealand Department of Statistics (September 2015: http://www.stats.govt.nz/browse_for_stats/economic_indicators/prices_indexes/FoodPriceIndex_HOTPSep15.aspx). Other data were shelf prices for house brands in the supermarket generally regarded as the cheapest in New Zealand. Where appropriate, a $NZ0.15/kg allowance is made for the cost of the simplest home cooking procedure required to generate a palatable, digestible product, by steaming, boiling or roasting.

As above, dietary energy and quality protein provision were determined from USDA data at http://nutritiondata.self.com/. No allowance is made for the potential impact of anti-nutritional factors, such as content of trypsin inhibitors in legumes, or indigestible peptide sequences in bread wheat.

Results are presented as ready-to-eat food intake (g/day) required to meet energy and quality protein needs. In some cases, the intake of protein required to meet all essential amino acid needs was less than 56 g. Consumption of that minimum intake would lead to a deficiency in dispensable amino acid intake. In those cases, the food intake necessary to consume 56 g/day of protein is used.

## Results and Discussion

All productivity estimates are given on a per-hectare basis, unless otherwise stated. In the milling wheat system, in which the wheat crop is followed by an autumn brassica crop to capture plant nutrients that would otherwise be lost to groundwater, flour production is sufficient to meet the energy needs of 26 people, and the protein needs of 16 people. Milk solids produced from milling offal and brassica dry matter meet the energy needs of an additional 8 people, and the protein needs of an additional 11 people. Thus, this baseline system is calculated to meet the energy needs of 34 people and protein needs of 27.

### Energy provision

Figure 1 shows that milk solids production from milling offal and a post-harvest brassica crop increases dietary energy yield in the milling wheat production system by 29%, while the grain maize production system produces 43% more dietary energy than milling wheat alone. Interestingly, production of field peas plus milk solids only achieves a 4% increase in dietary energy yield relative to milling wheat alone, due to the very low contribution of energy from the field peas.

This analysis supports the view that, in terms of dietary energy production, animal-derived foods are generally inefficient relative to cereal crops, although it can be seen (Fig. 1) that milk solids production from high-yielding silage crops is competitive with milling wheat in terms of the number of people whose dietary energy needs can be met from a hectare of prime arable land. A combination of maize silage plus short rotation ryegrass is projected to fulfil the energy requirements of about 25% more people than even the baseline system, in which milling wheat production is supplemented with a post-harvest brassica crop.

### Protein provision

Figure 2 shows that apart from beef production, all the animal food production systems outperform the baseline milling wheat in terms of the number of people whose protein needs are met from a hectare of prime arable land. In particular, milk solids production is a highly effective use of arable land to meet the requirements of humans for high-quality dietary protein.

**Table 2:** Essential amino acid composition of ideal protein and wheat and pea seed proteins.

Essential amino acid	Ideal content (mg/g protein)	Daily requirement (80 kg adult (mg))	Wheat (10.3%) (mg/g protein)	Peas (24.6%) (mg/g protein)
Tryptophan	6	336	12.33	11.18
Threonine	23	1,288	27.28	35.45
Isoleucine	30	1,680	34.66	41.22
Leucine	59	3,304	68.93	71.54
Lysine	45	2,520	22.14	72.03
Methionine + cysteine	22	1,232	39.03	25.37
Phenylalanine + tyrosine	38	2,128	80.78	74.92
Valine	39	2,184	40.29	47.11
Histidine	15	840	22.33	24.27

Cereals are the predominant sources of human foodstuffs, but are poor sources of protein: to obtain sufficient lysine from them, a considerable excess of dietary energy must be consumed. It has been suggested (Young & Pellett, 1994; Ghosh, Suri & Uauy, 2012; Day, 2013; Cassidy et al., 2013) that by combining ingredients derived from a number of plant sources, deficiencies in the protein quality of particular crop products can be corrected. Under the agroecological conditions described, the most productive crops are cereals and field peas. Table 2 gives the optimal levels in protein of the nine amino acids essential for human nutrition (United Nations University, 2007), and the essential amino acid composition of wheat flour (http://nutritiondata.self.com/facts/cereal-grains-and-pasta/5821/2) and split peas (http://nutritiondata.self.com/facts/legumes-and-legume-products/4353/2). In cereal-based diets, whether for humans or for monogastric animals, lysine is considered to be the first-limiting amino acid, and as can be seen (Table 2), legume protein appears to have this particular amino acid in excess relative to human requirement. Therefore, we estimated the optimal combination of flours from wheat and split peas needed to provide a mixture of proteins with ideal lysine content.

The deficit of lysine in white wheat flour can be corrected by consuming a mixture containing 54.2% wheat flour and 45.8% pea flour. Consumption of *ca.* 332 g of such a mixture will provide 56 g of protein, containing the daily requirement of lysine, but this quantity will only provide 49.4% of the daily requirement of phenylalanine and tyrosine. Thus, it is necessary to consume about 670 g of the wheat:pea mixture daily to ensure that needs of all essential amino acids are met, leading to the consumption of 113.4 g of protein. Such an excess of protein will be converted to dietary energy in the liver, and the quantity is well-below the safe upper limit for dietary protein intake (Bilsborough & Mann, 2006).

Using these figures, an independent calculation of the number of people whose nutrition needs can be met from 54.2% of a hectare of milling wheat, and 45.2% of a hectare of field peas was made: the energy demands of 21 people were met (as expected) whereas the protein requirements of 22 people were met, approximately 16% more than the geometric mean of the numbers fed by the individual crops alone. Thus, while the assertion is supported that mixtures of plant products can be better protein sources than any alone, they are well below the value of the animal protein that can be produced from the same area, since that area, devoted to producing milk solids, could meet the protein needs of 62 people.

### Limitations on seed protein quality

It is worth briefly considering the reason for this. The majority of plant food sources produced from prime arable land are the seeds or storage organs of a range of crop species. The endosperm in cereal seeds and the cotyledons of legume seeds have evolved to store plant nutrients for the use of the developing seedling after germination, but before the new plant is able to photosynthesise, acquire mineral nutrients from the soil, and in the case of the legumes, to nodulate and support nitrogen fixation by symbiotic microflora.

Vascular plants are able to synthesise their requirements of all the amino acids found in protein from fixed carbon and nitrate nitrogen, which may be derived from the amino acids in storage protein of any composition.

The essential amino acids are the most chemically-active found in protein, and are often part of the active site of enzymes, or involved in forming and stabilising the three-dimensional structure of biologically-active proteins. Lysine, in particular, is able to take part in the Amadori reaction with free carbonyl groups, forming condensation products which interfere with normal cytoplasmic biochemistry, and prevent the use of the lysine in protein biosynthesis. Consequently, it is not surprising that the content of lysine in storage proteins such as glutenins and gliadins is so low (Rombouts et al., 2009). Such lysine as is found in the wheat endosperm is likely to be associated with the small number of bioactive proteins present (but inactive) in the dormant seed, ready to take part in the necessary seed respiration prior to germination.

Similar considerations apply to the composition of the protein of the legume cotyledon. In this case, the level of lysine is relatively high, whereas the sulphur amino acids are poorly represented (Table 2). This means that unlike cereal protein, the first-limiting amino acids in legume protein are methionine and cysteine. The different ratio of lysine to sulphur amino acids between cereals and legumes is probably due to the presence of high levels of trypsin inhibitors in legume cotyledons. Legumes have evolved to produce substantial quantities of protein with trypsin-inhibiting properties (Savelkoul, Van der Poel & Tamminga, 1992) as a defence against pests. A wide range of other anti-nutritional factors are also present in the storage organs of plants used for human and animal feeding (Mann & Coles, 1998), further limiting the biological value of most plant proteins.

Nevertheless, the majority of the protein in the legume cotyledon is deposited to meet the nitrogen requirements of the developing seedling, and consequently has, generally speaking, the same bias against the most chemically-active amino acids in such storage protein. It is not surprising, therefore, that all plant seed storage proteins contain an excess of dispensable amino acids relative to the monogastric requirement for amino acid balance.

The search for mutants in cereals with more desirable seed amino acid composition has continued since the 1960s (Munck et al., 1970; Munck & Von Wettstein, 1974; Munck, 1970; Munck, 1972; Hard, 2002), but to date, there are no useful cultivars able to producing significantly-enhanced levels of essential amino acids in their storage proteins. Consequently, grain-based animal diets are often supplemented with industrially-produced pure amino acids. However, such amino acids are expensive, relative to animal protein sources, and their chemical activity means that during food or feed processing they are often irreversibly bound to other materials, meaning they are not available for a role in protein nutrition. Thus, it is improbable that combinations of plant seed storage protein and synthetic amino acids will ever be able to provide for human essential amino acid needs as efficiently as animal products.

### Optimal allocation of arable land to end use

**Figure 3:**
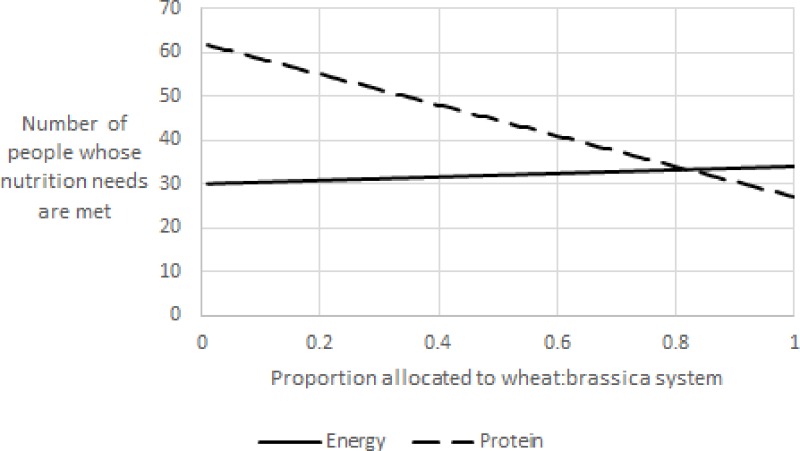
Estimation of optimal allocation of prime arable land to maximise the number of people fed (per hectare basis, meeting both energy and protein needs).

As can be seen above, direct use of plant products for food is generally the best allocation of arable land if dietary energy is the metric employed. However, we show that animal products are much more effective ways of delivering high yields of usable high-quality protein. This challenges the claims of those who argue that a global diet consisting entirely of plant-derived foods is the most efficient way to meet the dietary needs of the world’s population. Considerable discussion has already been devoted to the potential nutritional consequences of such a policy, and conversely, the means needed to improve livestock productivity (NAS Committee, 2015; Capper & Bauman, 2013; Pinstrup-Anderson, 2012; Smil, 2014; Wirsenius, Azar & Berndes, 2010). Clearly, then, there will be an optimal allocation of high-quality arable land to production of each nutrient. To illustrate this, we have estimated the best allocation of arable land based on two production systems: milling wheat with milk production from a post-harvest crop of brassica forage compared with production of milk solids alone from silage maize and an inter-crop of annual ryegrass. Figure 3 shows the number of people whose protein and energy needs are met from a hectare as the proportion of land allocated to each of those production systems is varied.

It is clear from this figure that the appropriate allocation to maximise the number of individuals for whom both protein and energy intake needs are met is approximately 82% to the wheat:brassica system. It should be noted that any bias should be in favour of the maize silage:annual ryegrass system, as a surplus of protein can be used to provide calories, whereas an energy surplus will not help meet a quality protein deficit. It should also be noted that the system meets the energy needs of just over 33 people from milk solids plus 5.25 tonnes of flour, or 435 g/person/day. That level of white flour consumption would provide just under 50% of the recommended daily intake of dietary fibre. It is beyond the scope of this paper to consider the intricacies of dietary fibre provision when resistant starch production during baking is considered as just one example.

### Economic considerations

Supplemental Information S2 (https://figshare.com/s/6d88301cbd122fd4c74f) complements the above analysis of the production of calories and proteins of high quality and analyses the daily cost of meeting energy and quality protein needs from a range of foods. These are divided into four categories (Table 3): meats, legumes, cereals and potatoes, and dairy (including eggs). Products were selected for inclusion provided there was both price and suitable nutrition information available.

**Table 3:** Calculation of daily cost of meeting dietary energy and protein needs. Price data from New Zealand metropolitan supermarkets. Amino acid value of egg protein = 1.00; prices are $NZ.

			Protein			To meet daily requirement (g of product as-is)		Cost/day to meet requirement ($NZ)		
Foodstuff (Ready-to-eat)	*Price/kg, $NZ*	*Energy (Kcal)*	*Gross (G/kg)*	*Amino acid value*	*Nett (g/Kg)*	*Energy*	*Protein*	*Energy*	*Protein*	*From 1st- limiting AA*
**Meats**										
Chicken, breast fillets	$11.79	1,650	310	1.33	412	1,455	136	$17.15	$1.60	$2.13
Ground beef	$13.43	2,460	240	0.67	161	976	348	$13.10	$4.68	$4.03
Corned silverside	$10.99	2,510	180	0.94	169	956	331	$10.51	$3.64	$3.42
Pork pieces	$18.99	3,760	140	1.50	210	638	267	$12.12	$5.06	$7.60
Whole chicken	$13.61	1,078	111	1.32	147	2,227	382	$30.31	$5.20	$6.87
Smoked frankfurters	$9.99	3,050	120	1.33	160	787	351	$7.86	$3.51	$4.66
Plain frankfurters	$8.99	3,050	120	1.33	160	787	351	$7.07	$3.15	$4.20
Smoked whole chicken	$9.99	1,650	180	1.33	239	1,455	234	$14.53	$2.34	$3.11
**Legumes as canned**										
Chilli beans	$7.00	1,120	60	1.09	65	2,143	856	$15.00	$5.99	$6.53
Baked beans	$5.80	940	60	0.71	43	2,553	1,315	$14.81	$7.62	$8.51
Butter beans	$3.80	1,430	90	0.96	86	1,678	648	$6.38	$2.46	$2.36
Lentils	$4.00	1,160	90	0.86	77	2,069	724	$8.28	$2.89	$2.53
Frozen peas	$2.25	780	50	0.84	42	3,077	1,333	$6.92	$3.00	$2.57
Chickpeas	$5.50	1,190	50	1.07	54	2,017	1,047	$11.09	$5.76	$6.16
**Cereal and potato**										
White bread	$1.82	2,660	80	0.52	42	902	1,346	$1.64	$2.45	$2.26
Breakfast biscuits	$5.45	3,730	110	0.52	57	643	979	$3.51	$5.34	$4.10
Rolled oats	$3.27	3,790	130	0.95	124	633	453	$2.07	$1.48	$1.41
Dry pasta	$5.65	3,710	130	0.45	59	647	957	$3.65	$5.41	$4.78
White rice	$2.44	1,300	30	0.71	21	1,846	2,629	$4.50	$6.40	$6.34
Potatoes	$1.80	1,980	40	1.09	44	1,212	1,284	$2.18	$2.31	$3.96
**Dairy**										
Whole milk (fresh chilled)	$1.67	640	30	1.37	41	3,750	1,363	$6.25	$2.27	$3.12
Whole milk powder	$8.19	4,960	260	1.37	356	484	157	$3.96	$1.29	$1.76
Cheddar cheese	$8.05	4,030	250	1.25	313	596	179	$4.79	$1.44	$1.80
Eggs	$6.27	1,420	130	1.37	178	1,690	314	$10.59	$1.97	$2.70

As expected, meats and dairy products (with the exception of butter: $2.09/day, not shown in main analysis, as it contains no dietary protein) were expensive sources of dietary energy, as were legumes, while starchy products (cereals and potatoes) were considerably cheaper. Table 4 provides means and variation for the cost of both daily energy and daily quality protein from each category. The cost of meeting daily protein requirement from a single foodstuff was calculated in two ways. Firstly, we determined the cost of providing the equivalent of 56 g of quality protein, by correcting for BV. This figure was not corrected for the impact of anti-nutritional factors often found in plant-derived foodstuffs such as legumes, as the cooking process reduces the impact of these. However, baking does not deal with the digestive inaccessibility of particular peptide motifs in wheat flour (N Larsen, pers. comm., 2016), so the cost of properly meeting protein needs from these foods is understated by an unknown amount.

**Table 4:** Mean cost of providing daily energy and protein from each food category. Prices are $NZ.

	Energy		Protein			
			*From BV estimate*		*From 1st -limiting amino acid content*	
	Mean	S.D	Mean	S.D.	Mean	S.D.
Meats	$14.08	$7.35	$3.65	$1.29	$4.50	$1.86
Legumes	$10.41	$3.84	$4.62	$2.12	$4.78	$2.63
Cereal and potatoes	$2.92	$1.12	$3.90	$2.05	$3.81	$1.77
Dairy	$6.40	$2.95	$1.74	$0.46	$2.35	$0.67

The second method of estimating the cost of providing for daily protein needs was based firstly on calculating the minimum quantity of the food required to supply the daily needs of essential amino acids, then, if this quantity did not provide the equivalent of 56 g of egg protein, increasing the quantity of food until this threshold was met. This approach thus ensures that the minimum cost either to supply all essential amino acids **or** the necessary total amount of protein is calculated for the analysis.

Employing either approach, we find that legumes are expensive sources of protein, with meat also costly. Among the meats, chicken is markedly cheaper than other sources, while lentils and frozen peas are cheaper protein sources than other legumes. The mean cost of protein from the cereals and potatoes group is higher than from meat, but rolled oats are a significant exception in this group, meeting daily protein needs for 40% lower cost than white bread, the next cheapest alternative. However, the cheapest way to meet protein needs is consumption of dairy foods. It is noticeable that the cost of protein from whole milk powder is only half that from fresh milk, presumably due to eliminating the need for continuous chilling of the product. Cheese also provides quality protein at the same low cost as whole milk powder. Since, as shown above, this class of foods is several-fold the most productive use of arable land, these results argue strongly for at least a proportion of total arable land to be used to produce dairy foods. It should be noted that while the conversion figures used are to produce an extra kg of milk solids, and represent best commercial practice (P Tocker, pers. comm., 2015), taking animal maintenance costs into account does not significantly lessen the dairy advantage, and new prebiotic technologies (Coles & Pearce, 2010; Coles & Pearce, 2011) will increase it.

While generally speaking, the contrasting approaches to calculating daily protein intake cost give similar results for the plant-based foodstuffs, the meat and dairy product daily costs are markedly higher when the calculations based on 1st-limiting amino acid content are compared to BV-based values. This is because these products have protein essential amino acid relative contents in excess of those required for ideal protein nutrition, and, accordingly, dispensable amino acid contents that are lower than can be sustained. Clearly, if low-cost protein sources were available that could complement the protein composition of these dairy and meat products, more cost-effective protein nutrition might be possible. Similarly, least-cost daily diets could be developed from a combination of these foods.

### Further considerations

The study reported here considers the optimum allocation of prime arable land (in this case, in a temperate, semi-maritime agroecological framework): land with the greatest flexibility of use for food production. It should be noted that much of the world’s agriculturally productive land falls outside this category, due to terrain that restricts mechanisation, or other agroecological considerations. Logically, agricultural productivity from such land, which is likely to be biased towards animal products, should be integrated with that from the prime arable land we consider. This may involve, for instance, beef and lamb finishing on arable land after the majority of growth has been achieved from forage on steep terrain. A significant use for such land in New Zealand is for the production of dairy heifer replacements.

A second consideration, not discussed above, is the land required for production overheads, such as seed for sowing, or feed for layer replacement breeders, broiler breeders or breeding sows. In a wheat production system, around 2.5% of the total land area will be used for seed production, with similar requirements for oats, brassicas, ryegrass and maize. Less than 1% of total feed use in poultry production is required for breeding stock, and at the end of their breeding life, the birds provide a further return of useful food. Breeding sows use about 3% of total feed put into a pork production system. Hence, there is little difference between production of plant-based and animal-derived foods with respect to production overhead land use.

We have not discussed root crops here. In the agroecological system described, root crops of importance in human nutrition include potato (*Solanum tuberosum*) and, to a lesser extent, Swede turnip (*Brassica napus*). Sugar beet (*Beta vulgaris*) is an important source of energy for human use, and protein and fibre for ruminant nutrition, but is not grown in New Zealand: cultivars developed specifically for animal use (as fodder beet) have a long history in this country, but pose many management problems, so are used sparingly. As a consequence, no data are available with which to make useful comparisons.

Potatoes are an important crop for the production of both table ware and raw materials for the production of processed foods. However, establishing the contribution they can make to food security in competition with other uses of arable land is very complex: there is a very wide range of management systems, and the practice of “ground-keeping” potatoes as a storage mechanism makes the question of whole-year land utilisation difficult to address. Furthermore, they must be managed in long-term rotations: at least ten years is recommended between crops in the same soil.

Nevertheless, according to the form of analysis used above, a single hectare of a main crop of potatoes producing 80 tonnes of table ware could, potentially, meet the energy needs of 70 people and the protein needs of 74 people annually. The individuals nourished this way would, however, have to consume more than 1.5 kg of potatoes/day—an intake only managed by rural Irish before the Great Famine of the mid-nineteenth century.

## Conclusions

The analysis described in this review clearly shows that prime arable land is capable of providing the most enhanced global food security from a carefully-selected mixture of uses aimed at production of both plant-based and animal-based foods. This complementation of food production systems is most important when focusing on meeting global need for high-quality protein, even if a crop such as potato is considered. The analysis shows that animal-based food production, particularly dairy production, can also make a critical contribution to food energy needs. This is particularly so when utilisation of biomass produced after a main crop is harvested, and before the next summer’s cropping programme begins, is considered. The cost-benefit analysis included supports this conclusion.

Clearly, the findings reported here cannot be generalised globally without further effort in data acquisition and deepened analysis. However, it is equally clear that the means to achieve global food security are more broad-based than earlier thought, and that humankind may enjoy a more varied diet in the future than has been feared.

## Additional Information and Declarations

### Data Availability

The following information was supplied regarding data availability: Figshare: https://figshare.com/s/6d88301cbd122fd4c74f. Figshare: https://figshare.com/s/ba9827a09ddb3396ce03.
